# Oral Health of Children and Adolescents in the United Arab Emirates: A Systematic Review of the Past Decade

**DOI:** 10.3389/froh.2021.744328

**Published:** 2021-09-29

**Authors:** Fatme Al Anouti, Myriam Abboud, Dimitrios Papandreou, Suzan Haidar, Nadine Mahboub, Rana Rizk

**Affiliations:** ^1^Department of Health, College of Natural and Health Sciences, Zayed University, Abu Dhabi, United Arab Emirates; ^2^Department of Public Health and Nutrition, College of Natural and Health Sciences, Zayed University, Dubai, United Arab Emirates; ^3^Department of Nutrition and Food Sciences, Lebanese International University, Beirut, Lebanon; ^4^Maastricht University, Maastricht, Netherlands; ^5^Institut National de Santé Publique, d'Épidémiologie Clinique et de Toxicologie (INSPECT-Lb), Beirut, Lebanon

**Keywords:** oral health, child, adolescent, United Arab Emirates, systematic review

## Abstract

Oral diseases are a universal public health problem with serious health and economic burdens. These diseases are a major concern in the pediatric population specifically. In the United Arab Emirates (UAE), among all the diseases that affect children, oral diseases, particularly early childhood caries, are the most common despite improvement in the provision of oral health services. Enhancing oral health status is one of the key public health goals in the country. This current systematic review aims to summarize the available data on oral health among children and adolescents in the UAE over the past decade (2011–2021). The review was conducted following a predefined protocol and in concordance with the Preferred Reporting Items for Systematic reviews and Meta-Analyses (PRISMA) statement. PubMed, the Cumulative Index to Nursing and Allied Health Literature (CINAHL) *via* EBSCO, EMBASE *via* Ovid, the Cochrane Library, and the Index Medicus for the Eastern Mediterranean Region (IMEMR) databases, and the gray literature were searched for original studies reporting on oral health in the pediatric population in the UAE, without applying any language restriction. Twenty-nine studies were included reporting on a total of 43,916 participants; they were mostly cross-sectional, and emirate-based; they were mostly limited by their setting and convenient sampling. Among the general pediatric population, results showed a high prevalence of dental caries across different emirates. Nevertheless, it was difficult to provide a predictable profile of caries, as risk factors were not well-explored and inconsistent across studies. Suboptimal oral hygiene practices were also prevalent, in addition to a low utilization of dental services. Furthermore, included studies showed a high level of oral problems in children with different disease (down syndrome, cerebral palsy, thalassemia, autism…) and special conditions (children in prison nurseries); yet, in general, treatment indices were lower than their healthy counterparts. This review suggests that dental caries is a major pediatric health problem in the UAE. Risk factors included higher consumption of snacks, being in public schools, lower maternal education level, and socioeconomic status. Nevertheless, risk factors were not well-explored and inconsistent across studies. Suboptimal oral hygiene practices and a low utilization of dental services were also identified, in addition to a high level of oral problems in children with different disease coupled with lower treatment indices in comparison with their healthy counterparts. This systematic review provides crucial information for planning and evaluating effective oral health programs, identifies gaps in the recent research in this field, and paves the way for preventive and interventional studies targeting oral health in pediatrics in the UAE. Immediate oral health promotion strategies are needed to address this public health problem early in its course by creating conditions that promote oral health, and increasing uptake of dental services. Intensifying research to draw temporal trends, understand the profile of childhood caries in the UAE, and explore cost-effective national community prevention programs are also needed.

## Introduction

Oral diseases encompass a wide range of conditions including dental caries, periodontal disease, tooth loss, and birth defects with dental disease associations such as cleft lip and palate [1]. These diseases affecting 3.5 billion people [2], and posing serious health and economic burdens [3] are undoubtedly a universal public health problem [3]. Fortunately, oral diseases are largely preventable [3], since their modifiable risk factors include mainly unhealthy diets high in simple sugars [1] within an environment of enamel adherent, acid-producing bacteria [4]. Furthermore, the evidence pinpoints an underlying influence of psychosocial, economic, environmental, as well as political factors in oral health, highlighting the importance of addressing underlying social determinants to promote sustainable oral health and reduce inequalities [5].

Oral diseases in the pediatric population are a major concern. Specifically, dental caries is the most common chronic disease in childhood [6], with global rates ranging from 12 to 98% among 4-year-old children [4]. Although the prevalence and severity of dental caries among 5–12-year-olds have declined over the last four decades, the decay-component remains very high [7]. While considered as none life-threatening, oral diseases are however associated with a wide range of clinical consequences amongst children, ranging from pain, discomfort, and lack of sleep [8], to negative impact on self-esteem, eating ability, compromised nutrition, and health [9], to increased absences from school and decreased academic performance [10], as well as decreased quality of life for both the children and their caregivers [3]. Furthermore, childhood caries experience is associated with increased risk of adulthood caries [11]. Oral health is thus a cornerstone for well-being, health, and quality-of-life in the pediatric population.

In high-income countries, the current approach to tackle oral diseases is trapped in a treatment-dominated, high-technology interventionist cycle that does not address the determinants of the disease [12]. Even with a decreased overall disease prevalence in the pediatric population, the progressive and cumulative nature of oral diseases into adult life remains a main issue [12].

In the United Arab Emirates (UAE), among all the diseases that affect children, oral disease, specifically early childhood caries, is the most common [13]. Available evidence indicates that, despite improvement in the provision of oral health services, dental caries remains a pediatric national health problem [14, 15]. Other oral diseases were seldom investigated; nevertheless, available evidence suggests a high prevalence of these diseases. For example, a national survey published in 2009 showed that only 37% of 15-year-olds schoolchildren had healthy periodontal tissues [15]. Enhancing oral health status is one of the key public health goals in the country [16, 17]. So far, numerous public health initiatives, such as the Community Dental Services (CDS)'s national oral diseases preventive program “Dubai Smiles Healthy” have been launched in schools and health centers with the aim of improving pediatric oral health in the UAE [18]. Additionally, the “Abu Dhabi Smiles” [16] is a school-based program launched in 2012 to provide awareness and advice on good oral hygiene practices for both parents and children whose ages range between 5 and 11 years [16]. The latest available literature review on this topic was published in 2014. It included studies published up to the year 2011, was limited to few databases, and focused only on dental caries in children younger than 13 years of age [14].

The current systematic review aims to summarize the available data on oral health among children and adolescents in the UAE over the last decade and hence could be valuable for policy and research considerations. Providing such information is crucial for planning and evaluating effective oral health programs. The review also aims to identify gaps in the recent research related to the pediatric oral health status in the UAE.

## Materials and Methods

### Protocol Registration

A predefined protocol for this systematic review was registered at the OSF registries (DOI 10.17605/OSF.IO/Y7PTZ).

### Criteria for Study Inclusion

In terms of Population, Exposure, Comparator, and Outcomes (PECO), our research question was defined as follows: P: pediatric population in the UAE, as well as their caregivers; E: including but not limited to sociodemographic and dietary factors, oral hygiene practices…; C: unspecified, such as poor dietary practices, suboptimal oral hygiene practices…; and O: including but not limited to prevalence and patterns of oral diseases, determinants and implications of poor oral health, oral hygiene knowledge and practices, utilization of dental services, as well as the effectiveness of programs and interventions aiming to improve oral health.

Hence, studies reporting on the national or Emirate-specific prevalence and patterns of oral diseases in children and adolescents, including dental caries, periodontal disease, tooth loss, birth defects with dental disease associations [1] were included. In addition, studies addressing the determinants of poor oral health, pertaining clinical, quality-of-life, and economic implications, oral hygiene knowledge and practices of children and their caregivers, utilization of dental services among healthy children and adolescents, and those with disease, and the effectiveness of programs and interventions aiming to improve oral health in the target population were also included.

Included studies had to be original articles (non-original studies such as editorials, case reports, case series, and reviews were excluded), address oral health in the pediatric population (i.e., in people aged <19 years), report data specific for UAE's citizens irrespective of their nationality (nationals and/or expatriates), sex (female or male), or health status and irrespective of the design (observational or interventional). Studies published after 2010 were included, without any language restriction.

### Search Strategy

The searches were run on PubMed, the Cumulative Index to Nursing and Allied Health Literature (CINAHL) *via* EBSCO, EMBASE *via* Ovid, the Cochrane Library, and the Index Medicus for the Eastern Mediterranean Region (IMEMR) databases. The bibliographies of included articles and previous relevant reviews were also hand-searched for eligible studies.

The search strategy considered three key terms: [1] United Arab Emirates, [2] children and adolescents, and [3] oral health. For each term, controlled vocabulary terms and text words were mapped. The electronic search strategy (Supplementary Material 1) was validated by a medical information specialist, and the search was run of May 4th 2021.

### Study Selection

One pair of authors (SH and RR) screened the titles and/or abstracts from electronic scientific databases using EndNote, version X6, and identified studies that potentially meet the inclusion criteria outlined above. Two pairs of authors (FA and RR; SH and NM) then screened the full texts of potentially eligible studies in addition to the records identified through the gray literature search. Calibration exercises were conducted before each step of this process; furthermore, study selection was conducted independently and in duplicate. Discrepancies were solved through discussions or with the help of a third reviewer.

### Data Extraction

Following a calibration exercise, two pairs of authors (FA and RR; SH and NM) extracted data from eligible studies using a data extraction form. This was done independently by each pair of authors and in duplicate, whereby discrepancies were solved through discussions or with the help of a third reviewer.

### Data Synthesis

A narrative synthesis of the findings from the studies was provided including the author-recorded characteristics of the study, details of the population included, as well as the study's methodology and main findings.

### Risk of Bias Assessment

Two pairs of authors (FA and RR; SH and NM) independently and in duplicate performed risk of bias assessment of the studies using the Newcastle—Ottawa Quality Assessment Scale adapted for cross sectional studies [19]. This scale assesses the appropriateness of representativeness of the sample, sample size, response rate, comparability in different outcome groups, and exposure and outcome assessment. Discrepancies between pairs of authors were solved through discussions or with the help of a third reviewer.

### Quality of Reporting

The authors followed the Preferred Reporting Items for Systematic reviews and Meta-Analyses literature search extension (PRISMA-S) checklist for the literature searching component of the systematic review [20], and the PRISMA statement for the reporting of the systematic review [21].

## Results

### Search Results

**Figure 1:**
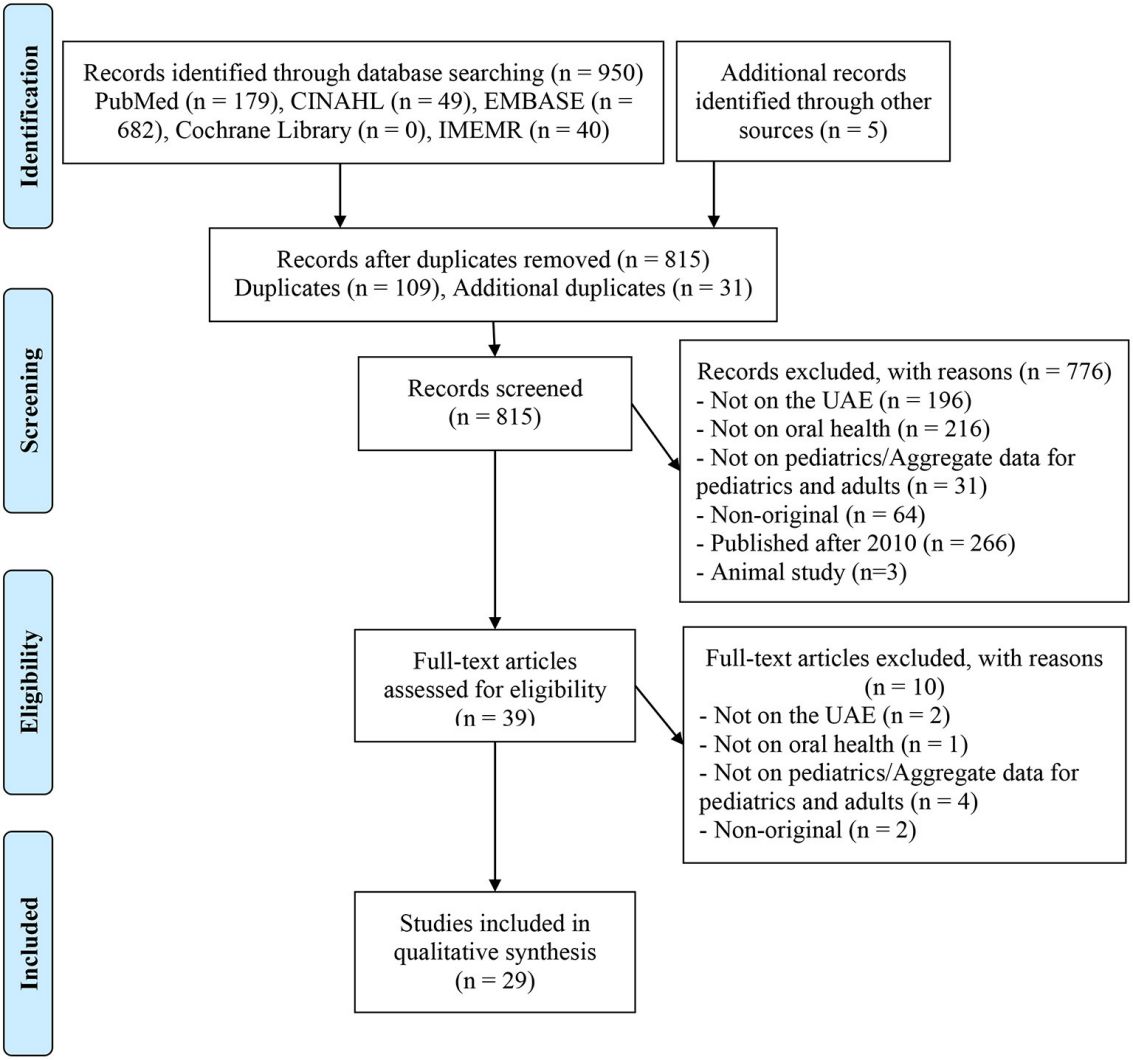
PRISMA diagram of study selection.

The details of the search process are detailed in Figure 1. A total of 29 studies were included in the systematic review [13, 22–49], reporting on a total of 43,916 participants.

### Characteristics of the Studies

Included studies were mostly cross-sectional [13, 22, 23, 25, 30, 31, 34–36, 38–47, 49], most of them (*n* = 10) took place in Dubai [22, 24, 25, 29, 31–33, 37, 42, 48], and only two were national [27, 49]. The sample size ranged between 54 [30] and 24,220 [49] participants. The populations studied included healthy preschool [13, 31, 35, 36, 38], school-aged [22–25, 34, 39–42, 45, 49], and secondary school children [44]. Also, included studies assessed preschool children of incarcerated mothers and their caregivers [27], preterm children [33], children diagnosed with leukemia [26], children with cerebral palsy [29], special health care needs children [30], beta thalassemia major children [32], children with down syndrome [37], children with autism spectrum disorder [43, 48], and mothers of preschool children [47].

The details of included studies (design, sample, methodology, results, and conclusion) are available in Supplementary Material 2.

### Outcomes of the Studies

Information related to caries' prevalence, indices, and risk factors from included studies are available in Table 1.

**Table 1:** Summary of caries-related data reported in included studies.

**References**	**Emirate, setting**	**Design**	**Population**	**Criteria**	**Prevalence**	**Indices**	**Risk factors**
**Preschoolers**							
AlKhayat [31]	Dubai, private preschools and kindergarten	Cross-sectional	Sample size: 2,957 Age group: 3–5 years %Males: 51.6% %Emirati: NR	WHO (1987)	38.5%	Mean(SD) dmft: 1.55(2.75) SIC: 4.55(3.12)	Caries prevalence and SIC index increased as the age increased NS gender-based differences
El Batawi and Fakhruddin [35]	Sharjah, day cares	Cross-sectional	Sample size: 435 Mean(SD) age: 1.2(3.6) years %Males: 55% %Emirati: 51.4%	WHO (2013)	22% in public day cares 77% in private day cares	Not assessed	NS difference between mean dmft score of the boys and of girls enrolled either in private or public daycare centers Sig. higher mean dmft score in residents enrolled in private daycares than nationals in public daycares (8.1 vs. 0.8) Sig. association between dmft scores and mothers' employment status in private centers (lowest scores among children of working mothers) Low dmft scores associated with high level of knowledge of caregivers in public centers
Elamin et al. [36]	Abu Dhabi, nurseries	Cross-sectional	Sample size: 186 Age group: 18 months−4 years; Mean age: 2.46 years %Males: 59.1% %Emirati: 46.2%	WHO (2013)	41%	Total sample: Mean(SD): dmft: 1.68(2.8); dt: 1.70(2.8); ft: 0.02(0.1); mt: 0 Emirati: dmft: 2.60(3.2); dt: 2.57(3.2); ft: 0.03(0.2); mt: 0 Non-Emirati: dmft: 0.75(1.8); dt: 0.75(1.8); ft: 0(0); mt: 0 SIC: Emirati: 50.8%; Non-Emirati: 15.9% Children residing in rural areas: 59.5%; Suburban: 36.5%; Urban: 12%	Sig. higher dmft and dt in Emirati children than non-Emirati Sig. higher dmft in Emirati children residing in rural areas than in urban or suburban areas NS difference in mean dmft between boys and girls SIC: Sig. higher among Emirati children than non-Emirati Sig. higher in children residing in rural areas NS difference between genders, or age groups Low maternal education, rural nursery location, infrequent tooth-brushing, frequent consumption of high-sugar food items and Emirati nationality were significantly associated with dental caries
Gopinath [38]	Sharjah, kindergarten	Cross-sectional	Sample size: 403 Age group: 5 years %Males: 48.1% %Emirati: 31.2%	WHO (1997)		Males: Mean(SD) dmft: 6.41(5.04); dt: 6.05(4.9); mt: 0.05(0.54); ft: 0.28(0.94) Females: Mean(SD) dmft: 6.01(4.95); dt: 5.61(4.88); mt: 0.19(1.20); ft: 0.24(0.84)	NS gender-based differences
Kowash et al. [13]	Ras Al Khaima, nurseries	Cross-sectional	Sample size: 540 Age group: 4–6 years; Mean(SD) age: 5.1(0.71) years %Males: 54.1% %Emirati: 100%	WHO (1997)	74.1%	Mean(SD) dmft: 3.07(0.13) (95% CI: 2.81–3.34) SIC: 13.3 (very high) Care index: 3.8% (very low)	NS association between time of tooth brushing, brushing time, frequency of sweet consumption, mother's dental knowledge and presence of caries
							Sig. association between frequency of visits to dentist, frequency of tooth brushing and presence of caries
Kowash [46]	Al Ain, Abu Dhabi, pediatric dentistry department	Cross-sectional	Sample size: 176 Age group: 1.5–5 years; Mean age: 3.7 years %Males: 57.3% %Emirati: 100%	BASCD criteria	99.4%	Mean: dmft: 10.9 (very high); dt: 10.2; mt: 0; ft: 0.7; dmfs: 32.1; ds: 30.3; ms: 0; fs: 1.8 Care Index: 6.4 % (very low) Restorative Index: 6.4% (very low)	Not assessed
**School-aged children**							
Ahmad et al. [22]	Dubai, governmental schools	Cross-sectional	Sample size: 779 Age group: 6–10 years; Mean(SD) age: 8.1(0.8) years %Males: 33.8% %Emirati: NR	EAPD criteria		Mean(SD) DMFT: Total sample: 2.41(1.70) (high) Low socioeconomic classes: 2.66(1.78) Middle class: 2.26(1.77) High class: 2.10(1.6)	Sig. higher in schools with low socioeconomic classes as compared to middle and high classes
Al Mashhadani et al. [25]	Dubai, public and private schools	Cross-sectional	Sample size: G1: 1,317 G2: 2,237 Age group: G1: 5–7 years G2: 12–14 years %Males: G1: 43.3% G2: 48.5% %Emirati: NR	WHO (2013)	G1: 60.3% diagnosed with decay 7.5% with missing teeth 21.6% with fillings ≈60% had untreated caries G2: 41.6% diagnosed with decay 7% with missing teeth 29.4% with fillings ≈41% had untreated caries	G1: dmft>0: 65.1% Mean dmft/DMFT: 3.87 G2: dmft>0: 59.2% Mean dmft/DMFT: 1.83	Not assessed
Gopinath et al. [39]	Sharjah, pediatric dentistry teaching clinic	Cross-sectional	Sample size: 405 Age group: 3–12 years; Mean(SD) age: 8.47(1.65) years %Males: 57% %Emirati: NR	WHO (1997)	Not assessed	≤ 6 years: mean(SD): dmft: Males: 7.86(3.35); Females: 6.97(3.38) DMFT: Males: 0.04(0.28); Females: 0.03(0.17) >6 years: mean(SD): dmft: Males: 6.00(3.55); Females: 5.79(3.84) DMFT: Males: 1.48(2.14); Females: 1.79(2.21)	Not assessed
Hashim et al. [40]	Ajman, public and private schools	Cross-sectional	Sample size: 1,036 Age group: 5–6 years %Males: 49.7% %Emirati: NR	WHO (1997)	s-ECC: 31.3% (95% CI: 23.6, 38.9)	Not assessed	Sig. associations between s-ECC and monthly income (OR: 1.43; 95% CI: 1.11, 1.85 for children from low-income families compared with children from high income families); high level of snack consumption (OR: 1.80 (1.26, 2.58) compared with using children with low snacking level); and dental visiting (OR for those visited a dentist because of a problem: 1.92 (1.49, 2.49) compared with those who had not visited the dentist in the previous year) NS association between s-ECC and age, gender, mother's education level, frequency of snacks between meals per day, and frequency of brushing
Hashim et al. [41]	Ajman, public and private schools	Cross-sectional	Sample size: 1,036 Age group: 5–6 years %Males: 50% %Emirati: 68.6%		Not assessed	Not assessed	Sig. associations between DMFT and frequency of snacking (aRR: 1.25; 95% CI: 1.00–1.57 for children who snacked 3 or more times daily compared with those who had snacked once daily), and level of snack consumption (aRR: 1.46; 95% CI: 1.26–1.70 for children with high snack consumption level compared with low consumption of snack), frequency of tooth brushing (aRR: 0.8; 95% CI: 0.64–0.93 for children who brushed their teeth ≥ twice daily compared with those who brushed their teeth less than daily) NS association between child and family characteristics and DMFT
**Secondary-aged children**							
Khadri et al. [44]	Sharjah, public/private schools	Cross-sectional	Sample size: 803 Age group: 11–17 years; Mean(SD) age: 12.8(1.4) years %Males: 50.4% %Emirati: 40.5%	WHO (1997)	Caries: 75.5% Decayed: 71.6% Missing: 4% Filled: 17.9%	Mean(SD) DMFT: 3.19(2.9)	Sig. association between DMFT and father's education level (−0.35; 95% CI: −0.53; −0.17), adolescent's age (0.42; 95% CI: 0.27–0.567), gender (0.41; 95% CI: 0.01–0.81), Arab ethnicity (0.74; 0.32–1.17), and soft drink consumption (0.31; 95% CI: 0.14–0.47)
Al Mashhadani et al. [25]	Dubai, public and private schools	Cross-sectional	Sample size: 2,063 Age group: 15–17 years %Males: 33.4% %Emirati: NR	WHO (2013)	42.4% diagnosed with decay 15.6% with missing teeth 39.3% with fillings 42.4% had untreated caries	dmft>0: 65.9% Mean dmft/DMFT: 2.70	Not assessed

*aRR, Adjusted Relative Ratio; BASCD, British Association for the Study of Community Dentistry; CI, Confidence Interval; dmfs, Decayed, Missing, and Filled Surfaces; DMFT, Decayed, Missing, and Filled Permanent Teeth; dmft, Decayed, Missing, and Filled Primary Teeth; DS, Down Syndrome; EAPD, European Academy of Pediatric Dentistry; G, Group; NR, Not Reported; NS, Not Significant; OR, Odds Ratio; SD, Standard Deviation; s-ECC, Severe Early Childhood Caries; SIC, Significant Caries Index; Sig, Significant; WHO, World Health Organization*.

#### Dental Caries

The majority of studies [13, 25, 31, 35, 36, 38–40, 44, 45] used the World Health Organization criteria for assessing caries. The prevalence of caries ranged from 22% in public day cares in Sharjah [35] to 99.4% in children attending a pediatric dentistry department in Al Ain, Abu Dhabi [46]. As for severity of caries, reported mean decayed, missing, and filled primary teeth (dmft) values ranged between 0.75 [36] and 10.9 [46]. Specifically, among Emiratis, mean dmft varied from 3.07 in children in Ras Al Khaima nurseries [13] to 10.9 in children attending a pediatric dentistry department in Al Ain, Abu Dhabi [46]. Only the study by Elamin et al. [36] conducted among children attending nurseries in Abu Dhabi presented data per nationality and highlighted significantly higher mean dmft indices in Emirati children than non-Emirati (2.60 vs. 0.75). Furthermore, this study showed significantly higher dmft values in Emirati children residing in rural areas than in urban or suburban areas [36]. Decayed, missing, and filled permanent teeth (DMFT) values ranged from 1.48 in healthy children attending a pediatric dentistry clinic in Sharjah [39] to 3.19 healthy secondary school children in Sharjah [44], with the decay component accounting for the majority of the DMFT values. No study presented data per nationality.

##### Risk Factors for Dental Caries

*Sociodemographic variables:* Majority of the studies reported a lack of significant association between dental caries and age [35, 36, 38, 40]. Regarding the association between the family's socioeconomic status and dental caries, Hashim et al. [40, 41] reported contradictory results; whereby Hashim et al. [40] found an inverse association between severe early childhood caries families' monthly income, whereas Hashim et al. [41] did not find a significant association between monthly income and DMFT. Moreover, Ahmad et al. [22] reported significantly higher DMFT values in schools with low socioeconomic classes. As for parental educational level, it was demonstrated that the mother's educational level was not associated with child's caries in the studies by Hashim et al. [40, 41], whereas father's educational level and DMFT values were negatively associated in one study by Khadri et al. [44].

*Dietary factors:* Soft drink consumption [44] and high extent of snacking [40, 41] were main dietary factors affecting dental caries. Interestingly, Kowash et al. [13] did not find a significant association between frequency of sweet consumption and dmft values.

*Oral hygiene practices:* Higher frequency of tooth brushing was a protective factor against caries in the study by Kowash et al. [13], and Hashim et al. [41]. A contradictory finding was reported by Hashim et al. [40]; also a non-significant effect of time of brushing was identified by Kowash et al. [13].

*Utilization of dental services:* Both Kowash et al. [13] and Hashim et al. [40] found that a higher frequency of dental visits was protective against caries.

#### Other Oral Diseases

Apart from caries, other assessed oral disease included dental erosion [38], molar-incisor hypomineralisation (MIH) [22, 42], fluorosis [22], tooth wear [23], and gingivitis [39, 44], the results of which along with the risk factors are presented in Table 2.

**Table 2:** Summary of oral-disease-related data reported in included studies.

**References**	**Emirate, Setting**	**Design**	**Population**	**Result**	**Risk factors**
**Preschoolers**					
Gopinath [38]	Sharjah, kindergarten	Cross-sectional	Sample size: 403 Age group: 5 years %Males: 48.1% %Emirati: 31.2%	Dental erosion: 58.8% Dissolution of enamel: 55.0% Exposed dentin: 3.7%	Predictors of dental erosion: Arab non-Emirati nationalities (OR: 0.27; 95% CI: 0.18–0.42); Caries experience (OR: 0.28; 95% CI: 0.16–0.51); Drinking sugary or carbonated beverages compared with water (OR: 0.30; 95% CI: 0.19–0.41)
**School-aged children**					
Ahmad et al. [22]	Dubai, governmental schools	Cross-sectional	Sample size: 779 Age group: 6–10 years; Mean(SD): 8.1(0.8) years %Males: 33.8% %Emirati: NR	MIH: 7.59% (low) Fluorosis: 10.9%, mostly very mild or mild	NS difference between genders (Male: 7.58%; Females: 7.57%) Prevalence of MIH: twice higher in low socioeconomic class schools (11.31%) as compared to high class (4.58%) Sig. higher mean DMFT in children with MIH than those without MIH [3.5(1.7) vs. 2.3(3.1); 49.5% of MIH cases had DMFT from 3 to 5 and 16.8% had DMFT ≥6] NS difference between genders, MIH status, and socioeconomic levels of school
Al Halabi et al. [23]	Abu Dhabi, schools	Cross-sectional	Sample size: 506 children; 9,213 teeth Age group: 2.6- 6.8 years; Mean(SD): 4.92(0.841) years %Males: 51.8% %Emirati: NR	Tooth wear: 97.6% Females: 49.39%; Males: 53.03% Severity in examined teeth: No tooth wear: 41.6%; Mild: 42.1%; Moderate: 16.2%; Severe: 0.15% Highest prevalence and severity of tooth wear in upper incisor and upper canine segments	Sig. associations between attrition and older age (95% CI: 1.07–2.06), mouth breathing (95% CI: 1.05–1.70), harder type of tooth brush (95% CI 1.03–1.69), mother's employment (95% CI: 1.14–1.88), and anterior deep bite (95% CI: 1.03–1.69)
Gopinath et al. [39]	Sharjah, pediatric dentistry teaching clinic	Cross-sectional	Sample size: 405 Age group: 3–12 years; Mean(SD): 8.47(1.65) years %Males: 57% %Emirati: NR	Plaque index: ≤ 6 years: Males: 1.63(0.75); Females: 1.33(0.74) >6 years: Males: 1.54(0.76); Females: 1.76(0.67) Gingival index: ≤ 6 years: Males: 1.06(0.81); Females: 1.06(0.79) >6 years: Males: 1.18(0.70); Females: 1.31(0.61)	Increase in dmft/DMFT values corresponds to increase in plaque index and gingival index scores Males were less likely to have gingivitis compared with females (OR: 0.47; 95% CI: 0.24–0.93), and children aged ≤ 6 years were less likely to have gingivitis than those aged >6 years (OR: 0.33, 95% CI: 0.17–0.62) NS association between tooth brushing frequency and plaque and gingival score values NS association between the physical structure of the diet with dmft/DMFT and plaque/gingival scores
Hashim et al. [41]	Ajman, public and private schools	Cross–sectional	Sample size: 1,036 Age group: 5–6 years %Males: 50% %Emirati: 68.6%	Mean(SD) plaque score: 0.67(0.32)	Children who had a high snack consumption level had higher mean plaque score than those with low snack consumption (adjusted difference: 0.13; 95% CI: 0.02–0.24) Children who brushed their teeth twice or more per day had lower mean plaque score than those who brushed their teeth less than daily (adjusted difference: −0.09; 95% CI: −0.17; −0.00) NS association between age, sex, mother's education, monthly income, snacks between meals per day, and plaque score Sig. association between plaque and DMFT (aRR for highest plaque category: 4.77; 95% CI: 3.67–6.19, compared with those in the lowest category)
					Children who snacked 3 or more times per day had higher mean DMFT and plaque than those who had snacked once per day (aRR: 1.19; 95% CI: 1.00–1.42) Children who had a high snack consumption level had higher mean DMFT and plaque than children with low consumption of snack (aRR: 1.21; 95% CI: 1.06–1.38) Children who brushed their teeth twice or more per day had lower mean DMFT and plaque than those who brushed their teeth less than daily (aRR: 0.85; 95% CI: 0.71–1.00) NS association between child and family characteristics and DMFT with plaque
Hussain et al. [42]	Dubai, public schools	Cross-sectional	Sample size: 342 Age group: 8–12 years; Mean(SD): 9.4(1.2) years %Males: 37.1% %Emirati: NR	MIH: 27.2% Females: 32.6%; Males: 18.1% Severity: mild: 53%; moderate: 17%; severe: 30% Incisor most frequently affected: Maxillary left central incisor (11.1%); and least frequently affected: mandibular left lateral incisor (0.6%)	High prevalence of MIH in school children, mainly with a mild severity Prevalence of MIH and MH was sig. higher in females, and related to location of tooth in the oral cavity
**Secondary-aged children**					
Khadri et al. [44]	Sharjah, public/private schools	Cross-sectional	Sample size: 803 Age group: 11–17 years; Mean(SD) age: 12.8(1.4) years %Males: 50.4% %Emirati: 40.5%	Plaque/gingivitis visible in at least 1 region of the oral cavity: 95% Plaque visible in all regions: 69.5% Unhealthy gums/gingivitis in all regions: 68.9%	Not assessed

*aRR, Adjusted Relative Ratio; CI, Confidence Interval; DMFT, Decayed, Missing, and Filled Permanent Teeth; dmft, Decayed, Missing, and Filled Primary Teeth; MH, Molar Hypomineralization; MIH, Molar-Incisor Hypomineralisation; NR, Not Reported; NS, Not Significant; OR, Odds Ratio; SD, Standard Deviation; Sig, Significant*.

Among 5 year-olds in Sharjah, Gopinath et al. [38] reported a high level of dental erosion (58.8%) and dissolution of enamel (55%). Predictors of dental erosion included Arab non-Emirati nationalities, caries experience, and consumption of sugary or carbonated beverages.

In school-aged children in Dubai, Ahmad et al. [22] and Hussain et al. [42] assessed MIH. The former reported a low prevalence of MIH (7.59%), which was mostly found in children attending low socioeconomic class schools, while the latter reported a higher prevalence reaching 27.2%, which was found especially in females (32.6%) compared with males (18.1%). When MIH was present, it was mostly mild (53%).

Al Halabi et al. [23] demonstrated a high prevalence of tooth wear in primary teeth (97.6%) among school children in Abu Dhabi. The authors also found the highest prevalence and severity of tooth wear to be at the level of the upper incisor and upper canine segments. Risk factors related to tooth wear included older age, mother's employment, mouth breathing, and anterior deep bite.

Gopinath et al. [39] reported high gingivitis and plaque scores among 3–12 year-olds attending a pediatric dentistry teaching clinic in Sharjah. Increase in dmft/DMFT values corresponded to increase in plaque and gingival scores. Also, the authors found that female gender and children more than 6 years were more likely to have gingivitis. No significant associations were identified between tooth brushing frequency and the physical structure of the diet, and plaque and gingival scores.

Hashim et al. [41] identified risk factors for plaque in 5–6 year-olds in Ajman, whereby high frequency of eating per day and high snack consumption level were both significantly associated with higher plaque scores. In contrast, children who brushed their teeth twice or more per day were found to have lower mean plaque score than those who brushed their teeth less than daily. The authors did not find any association between age, sex, mother's education, monthly income, snacks between meals per day and plaque score. The authors also suggested that children with high plaque scores were more likely to experience caries.

Finally, in secondary-aged children, Khadri et al. [44] identified a substantially elevated visibility of plaque/gingivitis in at least on region of the oral cavity, reaching 95% of the sample, as well as a high visibility of plaque in all regions (69.5%). The authors also reported a high prevalence of unhealthy gums/gingivitis in all regions (68.9%).

#### Knowledge and Practices

##### Children

Among preschoolers, Kowash et al. [46] reported a high level of poor oral hygiene reaching 63%, with 58% of the sample never or rarely brushing their teeth: 58%. In the same study, only 1 child out of the assessed 176 children was given fluoride.

In the recent national study conducted among a representative sample of school-aged children, Pengpid et al. [49] reported high levels of inadequate oral hygiene. In details, the prevalence of tooth brushing less than once daily mounted among males to 46.1% in 2016 without a change in comparison with values reported in 2010 (46.8%), nor in 2005 (48.6%). In females, although inadequate oral hygiene was relatively still high in 2016 reaching 28.3%; it reflected a significant reduction over time (37.9% in 2005, and 31.4% in 2010).

Conversely, Khadri et al. [45] reported high levels of daily tooth brushing reaching 93% among school-aged children in Sharjah. Hygiene practices were also found to be better among females, in terms of daily brushing (98 vs. 89%), brushing three times daily (19.6 vs. 13.8%), as well as tooth brushing at morning and evening (76.8 vs. 60.1%).

In his single-school study in Ajman, Dakhili et al. [34] reported good knowledge and practice regarding frequency of brushing, frequency of changing the brush, interdental cleaning, and cleaning the tongue. Nevertheless, the authors reported poor knowledge and practices regarding duration of brushing, method of brushing, use of mouthwash, frequency of tongue cleaning, and materials to clean the tongue were observed. Interestingly, a significant association was noted between correct knowledge and practices of dental hygiene, interdental cleaning and use of mouth wash, and tongue hygiene, as well as a significant positive correlation between knowledge and practice on oral hygiene.

Finally, only Khadri et al. [44] assessed oral hygiene practices among secondary-aged healthy children. The authors reported regularity in good hygiene in the sample, especially among females (97.5 vs. 89.4%). The percentage of children who brushed three times daily was low with highest value being reported for UAE nationals (22.8 vs. other Arabs: 14%; Indian subcontinent: 7.4%; Others: 16.9%). Also, more UAE nationals consumed fluoride supplements compared with participants from other nationalities (30.8 vs. other Arabs: 16.9%; Indian subcontinent: 8.1%; Others: 10.8%). Lastly, visiting the dentist in the past 12 months was low, and was least among Indian participants (25.2 vs. UAE nationals: 49.5%, other Arabs: 50.7%; Others: 53.8%).

##### Mothers

Knowledge, attitude, and practices of mothers were seldom assessed in the included studies. The study by Mahmoud et al. [47] among mothers of preschool children in Sharjah showed higher than average knowledge and excellent attitude toward their children's oral health, but mostly improper practices, and low levels of utilization of dental services, except during problems. Mothers' knowledge and practices were significantly associated with mothers' occupation and education. There was significant association between knowledge of mothers with their educational level; mothers with primary level of education had the highest scores, followed by those having a secondary education, then a university qualification, and finally by illiterate mothers. Also, practices of mothers were associated with their educational level; they were best among mothers with secondary level of education, and poorest among illiterate mothers. Interestingly, knowledge and attitude of mothers were associated with their occupation, with highest scores reported among employed mothers. Source of knowledge of mother's dental information included mostly relatives (27%), friends (23%), TV/Radio media (20%), reading (17%), and finally educational programs (13%).

Kowash et al. [13] explored the dental knowledge of mothers of Emirati preschool children in Ras Al Khaimah. The authors showed remarkable levels of poor knowledge among mothers of children with caries compared with mothers of caries-free children. For example, in that study, 13.7% of mothers thought that fluoride did not help in the prevention of tooth decay. Interestingly, 79% of those mothers had children with caries. Moreover, 3.5% of mothers did not consider that a balanced diet was important for the child's dental health and prevention of tooth decay; with 78% of those mothers had children with caries.

##### Caregivers

Only El Batawi and Fakhruddin [35] assessed caregivers in day care centers in Al Sharjah, and reported a significantly higher knowledge regarding oral health in those operating in public centers compared with private ones.

#### Utilization of Dental Services

Only the studies by Kowash et al. [13, 46] assessed the utilization of dental services among healthy Emirati preschoolers. The first study conducted in Abu Dhabi among children presented to a pediatric dentistry department [46] reported that two-thirds of children never visited a dentist, with very low care index and restorative index of 6.4%. Similarly, the second study [13], reported a very low care index of 3.8% among preschoolers in Ras Al Khaimah, despite the high mean dmft score of 3.07 reported by the authors.

#### Implications of Poor Oral Health/ or Dental Interventions

Khadri et al. [45] showed that school-aged healthy children with higher self-esteem scores brushed their teeth more often. The authors did not report any association between the presence of caries and self-esteem.

On the other hand, Alantali et al. [28] in the study conducted among preschool children showed that restorative dental general anesthesia resulted in significant improvement in child and family physical, psychological, and social aspects of quality-of-life, with a large change noted specifically in both the child and family impact sections.

#### Children With Disease/Special Conditions

Alnuaimi et al. [26] investigated the oral health of children with leukemia. The authors reported a 60% prevalence of oral problems, mostly consisting of oral mucositis and ulceration followed by dental caries and oral candidiasis. The prevalence of these problems was not associated with age, gender, nationality, nor family history; yet it was higher among patients who received treatment and follow-up locally within the country (75 vs. 2.8 in those treated abroad, and 22.2% treated in both locations). The peak occurrence of most oral problems was during phase IV (maintenance).

Al Hashmi et al. [29] explored oral health status among children with cerebral palsy in Dubai. More than half of the sample were diagnosed with caries (53%), with a mean(SD) DMFT index of 2.83(2.86), an oral health index of score of 1.68(1.34), and a 58.8% prevalence of gingivitis. These findings were similar to the age- and gender-matched control group. Patients with cerebral palsy presented a significantly higher calculus index [0.56(0.78) vs. 0.07(0.27)], as well as a higher rate of occlusal and oral soft tissues' anomalies, in addition to more a greater proportion and severity of erosion compared with healthy controls. Nevertheless, provision of services was low for cerebral palsy children, as exhibited by the restorative index (1.9), and was lower than the healthy counterparts (4.7), despite a higher treatment need, as exhibited by the met need index in the study group (0.32 vs. 0.24).

The oral health and problems of Emirati and non-Emirati children with β-thalassemia major in Dubai were explored by Al-Raeesi et al. [32], in comparison with healthy Emirati children. The overall prevalence of caries in children with thalassemia amounted to 68.4%, and the mean(SD) DMFT reached 1.49(2.67). Both findings were higher than values reported for the control group [48.7%; 0.21(0.56)]. Oral hygiene status was similar across study groups. More children with thalassemia had calculus than controls (28.9 vs. 7.9%), but less had gingivitis than controls (44.7 vs. 69.7%). More children with thalassemia than controls had gingival pigmentation (23.7 vs. 0%); no other differences were noted regarding soft-tissue anomalies and orofacial manifestations. Finally, the restorative care and treatment were lower in children with thalassemia.

Ghaith et al. [37] conducted a study on children with Down Syndrome. More than half of these children had dental decay (57.6%), and this rate was not different than that reported for healthy controls (57.6%). Yet, children with down syndrome had a higher mean(SD) DMFT [2.73(0.22) vs. 1.65(2.46)]. The oral health index score was similar between groups. This was also noted regarding the prevalence of gingivitis (65.4 vs. 70.4% in controls). However, the calculus index was higher among children with Down Syndrome [0.25(0.52)] compared with controls [0.07(0.27)]. Also, the prevalence of erosion (34 vs. 15.3%); severity of erosion (1.9 vs. 0%); prevalence of erosion into enamel (19.8 vs. 11.3%); and prevalence of erosion into enamel and dentine were higher among children with Down Syndrome (12.3 vs. 4%). Children with Down Syndrome had higher malocclusion problems, higher proportion of open bite, crossbite, scissor bite, anterior spacing, and posterior spacing, and more Class III molar relationship, in addition to a higher proportion of all oral soft tissues problems compared with healthy children. Nevertheless, children with Down Syndrome received more restorations and dental treatment and had better access to dental care (RI: 26.81 vs. 11.76; MNI: 35.6 vs. 23.6).

Both Jaber et al. [43] and Mansoor et al. [48] investigated children with Autism Spectrum Disorder. The first study [43] explored oral health status, and reported a prevalence of dental caries of 77% (males: 73.3%; females: 87.5). Mean(SD) dmft of the primary and early, mixed dentition years was: 2.2(1.77), and those of the late, mixed dentition and permanent dentition were 1.8(1.67) and 4.0(1.44), respectively. All children had gingivitis (generalized: 70.4%, or localized: 29.6%). The oral hygiene was mostly poor (59%), and finally, both the met need index and restorative index of the studied autistic children were low. Information regarding challenges to oral health among children with Autism Spectrum Disorder were provided by Mansoor et al. [48]. Regarding oral care at home, 83.3% reported that their children needed assistance in brushing their teeth, and 24.5% reported that their children always resisted tooth brushing. Around half of the parents reported that their children disliked the feeling of the toothpaste and toothbrush in his/her mouth (45.8 and 53%, respectively). These findings were higher than what was reported among healthy school-aged children. Around two-thirds of children with Autism Spectrum Disorder (65%) have visited a dentist, and this was not different than their healthy counterparts. Most common reason for not visiting a dentist was child being uncooperative, followed by child being afraid, having no complaint, and finally not finding a dentist who can handle the child. More than one-third (37%) of parents rated their child's experience as negative in the last dental visit, and 32.7% reported feeling more afraid or extremely afraid if their child had to go to the dentist tomorrow. Dislikes at the dentist included dentist drill, leaning back in the dental chair, loud sounds, bright light, and smell. Dislikes also included feeling of the toothbrush and toothpaste within mouth.

Finally, Alkhabuli et al. [30] assessed a group of Arab children with special healthcare needs in Ras Al Khaimah, and reported a high prevalence of dental caries (85.2%), especially among children with Down Syndrome and mental disability (62%), and a high mean(SD) dmft/DMFT [5.67(4.69)]. Around two-thirds of the sample had good oral hygiene (64.8%), without age- or gender-differences, but with differences across disabilities, where good oral hygiene was highest among children with autism (100%), and lowest in children with multiple disabilities (25%). Need for oral treatment was high, especially for restorations (89%), oral prophylaxis (41%), and orthodontic treatment (20%).

Alshehhi et al. [33] investigated oral health among preterm children when reaching an average age of 8, in comparison with a sample of full-term children. The authors reported a significantly higher mean(SD) DMFT [1.00(1.55) vs. 0.38(0.99)], and a 4 times higher prevalence of enamel defects (58.15 vs. 24.2%; OR = 4.33, 95% CI: 2.01–9.36) in preterm children, with the highest proportion found among those with abnormal birth weights. In addition to low birth weight, cesarean delivery and intubation were significantly related to the occurrence of enamel defects. In contrast, no relationship was reported between the prevalence of enamel defects and diseases during pregnancy, hospitalization in early life, systemic disease and antibiotic exposure in the first 3 years of life, and history of previous dental trauma. Finally, the most common type of enamel defects in the study group included white or creamy demarcated opacities, and post-eruptive breakdown.

In his case-controlled study, Al Salami et al. [27] reported a prevalence of caries of 89.8%, and a mean(SD) dmft score of 4.97(3.61) among children of incarcerated mothers. Both findings were similar to their counterparts. Nevertheless, the authors reported a worse oral hygiene (6.2%; 18.2%), a higher score of debris/plaque (93.8 vs. 81.8%), and a higher prevalence of calculus in the study group (3.1%; 0.4%). Utilization of dental services were lower in the study group, as exhibited by the lower restorative and care indices (4.2%; 4.43%, and 0.3%; 0.34%), despite a higher treatment need index in the study group (14.74%; 1.91%). The caregivers of prison nurseries showed unsatisfactory oral-health knowledge and attitude, especially knowledge on the effect of fluoride and its dental benefits, in addition to dietary habits. Knowledge and attitude of caregivers were in association, whereby 80% of caregivers with a satisfactory level of knowledge presented a positive attitude toward oral health while the level of the positive attitude of caregivers with poor knowledge reduced to 48%.

#### Oral Health Interventions

Al Mashhadani [24] investigated the effects of a 3-month school-based intervention consisting of daily tooth brushing with fluorinated toothpaste after mealtime among 1,500 students aged 4–6 years in 7 randomly chosen private and governmental schools. The intervention was multilevel. School nurses (or oral health coordinators) were trained to ensure proper tooth brushing technique, hygiene standards, and give oral health awareness tips to students, and equipped with special charts to follow up on students, and materials such as brushes and toothpaste. Also, parents were involved in the intervention, as they were introduced on the tooth brushing scheme, provided with information on healthy diet and good oral hygiene habits and tooth brushing, and charts to help follow up on the brushing at home. The evaluation based on a pre and post visible plaque index examination by dentists and dental hygienists, an interview with the involved school nurse, and a feedback questionnaire for the parents.

In post-intervention, the presence of visible plaque index decreased from 76.8 to 36.7%. Children accepted the activity, enjoyed participating daily, and had higher awareness of the importance of daily brushing and consequences of poor oral hygiene. The success of the activity increased when the school administration and nurse embraced and accepted having the students brushing daily in schools. Nevertheless, main barriers included: storage of the toothbrushes and maintenance of infection control standards; allowing students to leave the class for the activity; long-term cooperation of class teachers and administration. Parental feedback showed positive behavioral change toward tooth brushing (86%) with positive influence on siblings, established good oral hygiene routine (83%), increased interest to have more oral health sessions (72%), desire to have children continue to brush at school (79%), but a concern with infection control regarding storage of toothbrushes in schools (21%). This experience suggests that tooth brushing in schools might encourage and enforce good oral hygiene habits and improve children's oral hygiene status and the attitudes of their parents. This program showed promising results and could be implemented with the possibility of setting up policies and guidelines to govern its application in all schools.

### Risk of Bias Assessment

The detailed results of risk of bias assessment are available in Supplementary Material 2. The assessment of the outcome was optimal in the majority of the studies. Nevertheless, major shortcomings pertained to the representativeness of the sample and its limited size, as well as the absence of information related to the response rate.

## Discussion

The aim of this systematic review was to summarize the available data on oral health among children and adolescents in the UAE over the past decade in order to provide important information that could be utilized by stake holders for future planning of effective preventive and interventional program. The main findings shed light on the high prevalence of dental caries among pediatric population including those with special conditions and diseases across all emirates of the UAE. Moreover, oral hygiene practices and utilization of dental services were suboptimal and low.

Maintaining oral health and hygiene starting from an early age is crucial for maintaining well-being throughout life span. According to WHO and the American Academy of Pediatric Dentistry, the best time to for a child's visit to a dental care provider is at the age of 1-year-old past the emergence of the first tooth [50, 51]. The global prevalence of childhood dental caries- one of the most common oral diseases, has ranged between 12 and 98%, with about 600 million children being affected worldwide [4]. When children develop oral diseases like dental caries or gingivitis, this might be accompanied by pain, discomfort, lack of sleep, an inability to ingest food, and more visits to the dentists [8]. Consequently, this will interfere with their daily life activities, nutrient and food intake, energy levels and might even deter them from learning [8]. Moreover, Rebelo et al. [10] showed that untreated dental caries has an inverse relationship with school performance and attendance among children. According to Al-Bluwi [52], about 50 million hours of school is lost every year due to oral health issues. The burden of untreated oral diseases does not only impact the academic and social life of children, but also surpasses to financial tolls. For instance, in 2010, 298 billion US dollars were spent globally in treating dental caries [53].

Among all the diseases that affect children in the UAE, early childhood dental caries is considered the most common [13]. Recent updates about oral health status for children and adolescents in the UAE are lacking. A previous informative review on oral health among children in the UAE highlighted studies published up to the year 2011. However, the review was limited to few databases and focused only on dental caries in children younger than 13 years of age [14]. The current systematic review aims to first summarize the available data on oral health among children and adolescents in the UAE over the past decade and second to highlight gaps pertaining to this area and provide valuable implications for future policy and research considerations. Despite the significant advancement in dental services in the UAE, dental caries remains highly prevalent among children in the UAE. Although El Nadeef et al. [15] and Al-Bluwi [14] recommended to conduct routine epidemiological studies to assess oral health and understand its determinants, to the best of our knowledge, there are no new nation-wide studies to underscore the prevalence of caries and DMFT among children and adolescents. Being a country with multicultural diversity poses a major challenge to research aiming to explore risk factors and intervention-based treatments [14].

The high prevalence of dental caries documented in our study is similar to the results reported by researchers from the Eastern Mediterranean Region, reaching among 12-year-olds a prevalence of 70% in Bahrain, 62% in Iraq, 86.9% in Lebanon, and 90.2% in Yemen [54].

The only comprehensive national survey of oral health of children in the UAE conducted by El Nadeef et al. [15] determined the prevalence of dental caries among 12 and 15-year-old children (*N* = 2,651) indicated 54 and 65% prevalence, respectively [15]. The results were comparable to those reported by researchers from the neighboring country Saudi Arabia, indicating a prevalence of approximately 80% for dental caries among Saudi children for primary teeth and 70% for permanent teeth [55].

The study by Elamin et al. [36] investigated the influence of socioeconomic factors, oral hygiene practices and eating habits on the prevalence of dental caries in preschool children living in Abu Dhabi. Lower maternal educational levels along with lack of regular tooth brushing and elevated intake of sugar rich foods were the most prominent risk factors. Moreover, Emiratis had a more significant prevalence of dental caries as compared with non-Emiratis [36]. These results highlighted the need for effective interventions to improve dental habits in children and regular dental screenings for this age group. The high intake of sugary foods and drinks being readily available and affordable to purchase in the UAE for both Emirati and non-Emirati children warrants more investigation before conclusive results are inferred about the socioeconomic status. The contradictory results reported by Hashim et al. [40, 41] and Ahmad et al. [22] regarding the relation between the family's socioeconomic status and dental caries could be due to the distinctive socioeconomic factors of the country since income alone does not determine the socioeconomic level in the UAE.

Our results demonstrate a high prevalence for dental caries among both children and adolescents in the UAE. The study by Elamin et al. [36] highlighted significantly higher prevalence of dental caries among Emirati children as compared with non-Emirati (dmft 2.60 vs. 0.75). This was the only study that discerned differences in pediatric oral health status based on nationality, hence solid conclusions cannot be inferred. Nevertheless, the findings might be attributed to the more frequent checkups and availability of subsidized dental health services to UAE nationals. Ironically, Emirati children especially in rural areas have a tendency to consume traditional foods whereas expatriate non-Emirati children tend to consume more fast food and sweetened snacks. Also, the difference in access of dental care and possibly insurance coverage between the two groups should be noted. In general, specific risk factors implicated with higher dental caries scores were higher consumption of snacks, being in public schools, lower maternal education level, and socioeconomic status. Specifically, parents of higher socioeconomic status have a higher level of oral health awareness and tend to be aware about the importance of early dental checkups [56]. Contradicting results however were revealed by other researchers for children with higher income families who were shown to have higher scores for dental caries. In addition, a robust correlation existed between knowledge and practice in different areas such as brushing techniques and flossing [34]. Mahmoud et al. [47], later demonstrated that interestingly the levels of knowledge and attitude of the interviewed mothers were sufficient, but their practices concerning their children's oral health were inappropriate. The results reported by Mahmoud et al. [47] regarding the high knowledge of mothers with primary level of education is worth further investigation. This association should be explored in the context of other factors including the mother's occupation, age, employment, source of knowledge about dental information and socioeconomic status. In addition, the presence of an educated care giver in the house as is common with many Emirati families might play a major role in this sense by offering information and resources about dental hygiene and oral health for the children.

Abu Gharbieh et al. [56] reported significant differences in the oral health knowledge score in terms of age, gender, and nationality. Emirati parents showed a higher level of knowledge about their children's oral health as compared to non-Emirati parents. The researchers suggested that the significant prevalence of diabetes mellitus among Emirati parents might have played a role in increasing the level of awareness about oral health and hygiene in general since these individuals receive more frequent educational information during medical visits to health professional to manage their diabetic condition. Moreover, the researchers concluded that females were more knowledgeable and practicing better oral health behavior than males.

On a relevant context, a more recent study by Pengbid et al. [49] demonstrated the presence of inadequate oral hygiene knowledge and practices among adolescents in a cross-sectional national survey in 2005, 2010 and finally 2016 as part of the UAE Global School-Based Student Health Survey (GSHS). This highlights the importance of school-based interventions in improving knowledge and practice and the need for public health professionals to focus on specific factors when designing oral health educational programs to increase levels of knowledge and help individuals develop healthy oral habits. In addition, it is imperative to carefully design oral health education programs to include not only parents but also school teachers and care givers especially that home caregivers could be domestic helpers recruited from abroad and hence might have cultural gaps.

One particular category pertains to children with special conditions and diseases. There is a high level of oral problems in children with different disease (down syndrome, cerebral palsy, thalassemia, autism) and special conditions (children in prison nurseries); yet, in general, treatment indices are lower [26, 29, 37, 43, 48]. Despite the availability of services, utilization level by children with different diseases remains low. This might be attributed to the fact that such services might require more specific tailoring to match the needs of children with special conditions.

The implications of our study could guide future intervention studies which are very limited and lacking. The role of public health professionals in conducting educational programs to not only raise awareness but to ensure that knowledge is indeed translated into practice is of utmost importance.

The main focus of this review was on dental caries as a major and highly prevalent oral health disease, however the high prevalence of other oral health diseases is documented by few researchers. Although such studies are very heterogeneous and limited in number, the contributions of other diseases like dental erosion [38], fluorosis [22], tooth wear [23], and gingivitis [39, 44] to the scope of oral health among children and adolescents in the UAE should not be ignored. While the prevalence of dental erosion was associated with the consumption of sugary carbonated beverages, the prevalence of tooth wear was correlated with mother's employment and mouth breathing. Moreover, frequent snacking was linked to plaque development among children and adolescents as this prolonged exposure to food remains within the mouth.

It is worth mentioning that most included studies emanated mainly from the largest emirates within the country: Abu Dhabi, Dubai, and Sharjah. Additional research from other emirates is urgently needed. Moreover, a solid predictive model with significant risk factors for caries might be very challenging because of the vast differences within the UAE as a country with a multicultural background [14]. Finally, the results of some studies cannot be extrapolated to the target population because of using specific conditions such as limited setting and convenient sampling.

### Strengths and Limitations

To our knowledge, this is the first review to systematically explore the epidemiology of oral health among healthy children and adolescents and those with special conditions in the UAE over the past decade. This review was conducted according to a pre-defined protocol and following standard methods for reporting systematic reviews [20, 21]. We searched numerous databases, as well as the gray literature to increase the exhaustiveness of the search. Yet, as for all systematic reviews, this quality of evidence of this work is inherently limited by the design and quality of included individual studies, whereby the majority of the studies were conducted in only one Emirate, and or limited by their convenient sampling technique, bivariate statistical analysis methods, and generalizability of their results. Within this scope, as the included studies were of significant heterogeneity in terms of exposure and outcome assessment methods, and also study population, we were not able to meta-analyze available data especially those related to the dental caries indices or factors contributing to the development of dental caries and other oral diseases. Nevertheless, our work holds important implications for dental health policy in the UAE. By focusing on modifiable risk factors in the pediatric population and utilization of dental health services in this population, this review can guide informed dental public health policies, and subsequently targeted prevention and intervention programs, thereby decreasing inequities in dental health. Finally, by mapping existing epidemiological and interventional studies, this review highlights the gaps in pertaining research in the country. Research should be intensified to draw temporal trends and understand the profile of childhood caries in the UAE, as well as to explore cost-effective national community prevention and intervention programs.

## Conclusion

Since the oral cavity is the first station of the digestion process, where different types of food and drinks are ingested, it is important to maintain its hygiene and health starting from an early age using the correct methods. The multifactorial nature of the oral health diseases among children makes the theoretical pathways that link the relationships among predictor variables and risk factors with oral health outcomes children very complex. Specifically, biological pathways play crucial roles in susceptibility and resistance to this complex disease. These factors include most importantly the dietary features, i.e., cariogenic potential of foods (e.g., sucrose), the frequency of eating, and the physical state of the diet, which can affect individually or jointly the carious process. Other physiological factors, include salivary flow and composition especially in calcium and phosphates, tooth morphology, whereby malposition of the teeth or deep anatomy grooves can hinder tooth brushing, and fluoride penetration, as well as enamel and dentin formation processes, which are genetically-regulated through interactions with oral bacteria to affect caries susceptibility and/or enhancement of the enamel thickness/fluoride concentration, hence protecting teeth against caries [57].

Within the limitations of this review, it can be suggested that dental caries is still a major pediatric health problem in the UAE. Suboptimal oral hygiene practices, coupled with a low utilization of dental services, were reported. Moreover, a high level of oral problems in children with different disease was noted; yet, in general, treatment indices in these patient populations were lower than those of their healthy counterparts. Accordingly, immediate oral health promotion strategies are needed to address this public health problem early in its course by creating conditions that promote oral health, and increasing uptake of dental services. Our findings echo the call of Al Mashhadani [24] for a mandatory law requiring a dental health clearance (status and certification) for student registration, a referral system for students requiring immediate dental care, and a policy to enforce supervised tooth brushing in schools [24]. Ongoing culturally-sensitive and practical awareness and education programs are also needed to change the behavior of parents or caregivers, toward enhanced oral health practices in children. Finally, more studies reporting on national dental caries and exploring determinants of this disease are needed to obtain an accurate picture of pediatric dental caries in the country.

## Data Availability Statement

The original contributions presented in the study are included in the article/Supplementary Materials, further inquiries can be directed to the corresponding author.

## Author Contributions

FA and RR were involved in the concept and design. SH performed the searches. SH and RR conducted the title and abstract screening. FA, RR, SH, and NM conducted the full text screening, performed the data extraction, and risk of bias assessment. All authors contributed to writing the draft manuscript and have read and agreed to the published version of the manuscript.

## Funding

This study was funded by the cluster grant R18030 awarded by Zayed University, United Arab Emirates. The funding body was not involved in the design of the study and collection, analysis, and interpretation of data or in writing the manuscript.

## Conflict of Interest

The authors declare that the research was conducted in the absence of any commercial or financial relationships that could be construed as a potential conflict of interest.

## Publisher's Note

All claims expressed in this article are solely those of the authors and do not necessarily represent those of their affiliated organizations, or those of the publisher, the editors and the reviewers. Any product that may be evaluated in this article, or claim that may be made by its manufacturer, is not guaranteed or endorsed by the publisher.

## Supplementary Material

Supplementary Material 1 Search Strategy.

Supplementary Material 2 Details of included studies.
