# Supplementary material for: Oral Health of Children and Adolescents in the United Arab Emirates: A Systematic Review of the Past Decade
Source: Front Oral Health. 2021 Sep 29;2:744328. doi: 10.3389/froh.2021.744328 (PMC8757796; doi:10.3389/froh.2021.744328)
Supplement: Supplementary Material 1 — Search Strategy. [file Table_1.DOC]

**Identification**

Records identified through database searching (n = 950)

PubMed (n = 179), CINAHL (n = 49), EMBASE (n = 682), Cochrane Library (n = 0), IMEMR (n = 40)

Additional records identified through other sources (n = 5)

Records after duplicates removed (n = 815)

Duplicates (n = 109), Additional duplicates (n = 31)

**Screening**

Records screened
(n = 815)

Records excluded, with reasons (n = 776)

- Not on the UAE (n = 196)

- Not on oral health (n = 216)

- Not on pediatrics/Aggregate data for pediatrics and adults (n = 31)

- Non-original (n = 64)

- Published after 2010 (n = 266)

- Animal study (n=3)

**Eligibility**

Full-text articles assessed for eligibility
(n = 39)

Full-text articles excluded, with reasons (n = 10)

- Not on the UAE (n = 2)

- Not on oral health (n = 1)

- Not on pediatrics/Aggregate data for pediatrics and adults (n = 4)

- Non-original (n = 2)

**Included**

Studies included in qualitative synthesis
(n = 29)

CINAHL: Cumulative Index to Nursing and Allied Health Literature; IMEMR: Index Medicus for the Eastern Mediterranean Region; UAE: United Arab Emirates
