# Supplementary material for: Oral Health of Children and Adolescents in the United Arab Emirates: A Systematic Review of the Past Decade
Source: Front Oral Health. 2021 Sep 29;2:744328. doi: 10.3389/froh.2021.744328 (PMC8757796; doi:10.3389/froh.2021.744328)
Supplement: Supplementary Material 2 — Details of included studies. [file Table_2.DOCX]

Supplementary Material 1: Search Strategy

**PubMed**

| Search number | Query |
| --- | --- |
| 28 | #22 and #26 and #27 |
| 27 | ((pediatric* or paediatric* or child* or newborn* or congenital* or infan* or baby or babies or neonat* or pre-term or preterm* or "premature birth*" or NICU or preschool* or pre-school* or kindergarten* or kindergarden* or "elementary school*" or "nursery school*" or ("day care*" not adult*) or schoolchild* or toddler* or boy or boys or girl* or "middle school*" or pubescen* or juvenile* or teen* or youth* or "high school*" or adolesc* or pre-pubesc* or prepubesc*) OR child*[journal] or adolesc* [journal] or pediat*[journal] or paediat*[Journal] OR child[MeSH Terms] OR infant[MeSH Terms] OR child, exceptional[MeSH Terms] OR adolescent[MeSH Terms] OR pediatrics[MeSH Terms] OR child, abandoned[MeSH Terms] OR child, orphaned[MeSH Terms] OR child, unwanted[MeSH Terms] OR minors[MeSH Terms] OR ("congenital, hereditary, and neonatal diseases and abnormalities"[MeSH Terms])) |
| 26 | #23 or #24 or #25 |
| 25 | Dubai[tw]OR abudhabi[tw] OR abu dhabi[tw] OR ajman[tw] OR fujaira*[tw] OR sharja*[tw] OR sharga*[tw] OR sharka*[tw] OR ras alkhaima*[tw] OR ras elkhaima*[tw] OR ras al khaima*[tw] OR ras el khaima*[tw] OR umm al qaiwain[tw] OR umm el qaiwain[tw] OR umm al quwain[tw] OR umm el quwain[tw] OR umm alqaiwain[tw] OR umm elqaiwain[tw] OR om alqaiwain[tw] OR om elqaiwain[tw] OR umm alqaiwain[tw] OR umm elqaiwain[tw] OR om alqaiwain[tw] OR om elqaiwain[tw] OR umm alquwain[tw] OR umm elquwain[tw] OR om alquwain[tw] OR om elquwain[tw] |
| 24 | Emirat*[tw] OR UAE[tw] OR bedouin*[tw] OR gulf[tw] OR trucial state*[tw] |
| 23 | United Arab Emirates[mesh] OR Arabs[mesh] |
| 22 | #1 or #2 or #3 or #4 or #5 or #6 or #7 or #8 or #9 or #10 or #11 or #12 or #13 or #14 or #15 or #16 or #17 or #18 or #19 or #20 or #21 |
| 21 | oral health[tw] or mouth disease*[tw] or oral hygien*[tw] or tooth disease*[tw] or tooth abnormal*[tw] or tooth wear*[tw] or tooth injur*[tw] or tooth loss*[tw] or tooth resorption[tw] or dental carries[tw] or tooth demineralization[tw] or periodontal disease*[tw] or stomatognathic disease*[tw] or dental pulp disease*[tw] or toothache*[tw] or plaque[tw] |
| 20 | dental hygiene[tw] or dental pulp exposure*[tw] or dental disease*[tw] or tooth abrasion*[tw] or tooth attrition*[tw] or dental wear*[tw] or tooth fracture*[tw] or tooth avulasion*[tw] or teeth injur*[tw] or dental carries[tw] or tooth hypomineralization[tw] or teeth hypomineralization[tw] or carious lesion*[tw] or root carries[tw] or dental fissure*[tw] or tooth mobilit*[tw] or tooth migration*[tw] or malocclusion*[tw] or tooth crowding*[tw] or cross bite*[tw] or oral health status[tw] or tooth birth defect*[tw] or dentistry[tw] or cleft lip[tw] or dental health service*[tw] or oral health program*[tw] or hair lip*[tw] |
| 19 | Dental Plaque[mesh] |
| 18 | toothache[mesh] |
| 17 | Dental health services[mesh] |
| 16 | cleft lip[mesh] |
| 15 | dental pulp diseases[mesh] |
| 14 | stomatognathic diseases[mesh:noexp] |
| 13 | malocclusion[mesh] |
| 12 | periodontal disease[mesh] |
| 11 | tooth demineralization[mesh] |
| 10 | dental caries[mesh] |
| 9 | tooth resorption[mesh] |
| 8 | tooth loss[mesh] |
| 7 | tooth injuries[mesh] |
| 6 | tooth wear[mesh] |
| 5 | tooth abnormalities[mesh] |
| 4 | tooth diseases[mesh:noexp] |
| 3 | oral hygiene[mesh] |
| 2 | mouth diseases[mesh:no exp] |
| 1 | oral health[mesh] |

**EMBASE via Ovid**

| **#** | **Query** | **Results from 3 May 2021** |
| --- | --- | --- |
| 1 | exp oral health/ or mouth diseases/ or exp oral hygiene/ or tooth diseases/ or exp tooth abnormalities/ or exp tooth wear/ or exp tooth injuries/ or exp tooth loss/ or exp tooth resorption/ or exp dental caries/ or exp tooth demineralization/ or exp periodontal disease/ or exp malocclusion/ or stomatognathic diseases/ or exp Dental Plaque/ or exp toothache/ or exp Dental health services/ or exp cleft lip/ or exp dental pulp diseases/ | 1,127,285 |
| 2 | ((tooth or teeth or dental or oral or mouth) adj3 (health or disease? or hygien* or abnormalit* or wear* or injur* or loss or resorption? or fractur* or (birth adj defect?) or fissure? or anomalies or malformation* or fused)).mp. | 188,381 |
| 3 | ((periodental or stomatognathic) adj3 disease?).mp. | 191 |
| 4 | (toothache* or dentistry or malocclusion* or (cleft adj lip?) or (cross adj bite?) or (hair adj lip?) or (root adj caries) or (carious adj lesion?)).mp. | 127,546 |
| 5 | ((dental or tooth or teeth) adj2 (exposure? or abrasion* or attrition* or avulasion* or caries or demineralization? or hypomineralization* or plaque or migration* or crowding? or mobilit*)).mp. | 74,921 |
| 6 | 1 or 2 or 3 or 4 or 5 | 1,220,042 |
| 7 | juvenile/ or exp adolescent/ or exp child/ or exp postnatal development/ or (pediatric* or paediatric* or child* or newborn* or congenital* or infan* or baby or babies or neonat* or pre term or preterm* or premature birth or NICU or preschool* or pre school* or kindergarten* or elementary school* or nursery school* or schoolchild* or toddler* or boy or boys or girl* or middle school* or pubescen* or juvenile* or teen* or youth* or high school* or adolesc* or prepubesc* or pre pubesc*).mp. or (child* or adolesc* or pediat* or paediat*).jn. | 4,962,868 |
| 8 | exp United Arab Emirates/ or exp Arabs/ | 11,775 |
| 9 | (emirat* or UAE or bedouin* or gulf or (trucial adj state*)).ti,ab,sh. | 24,594 |
| 10 | (dubai or abudhabi or (abu adj dhabi) or ajman or fujaira* or sharja* or sharga* or sharka* or (ras adj (alkhaima* or elkhaima* or ((al or el) adj khaima*))) or ((um* or om*) adj (((al or el) adj (qaiwain or quwain)) or alqaiwain or alquwain or elqaiwain or elquwain))).ti,ab,sh. | 2,054 |
| 11 | 8 or 9 or 10 | 32,487 |
| 12 | 6 and 7 and 11 | 682 |

**CINAHL via EBSCO**

| # | Query |
| --- | --- |
| S62 | MH oral health+ |
| S61 | MH mouth diseases |
| S60 | MH oral hygiene+ |
| S59 | MH tooth diseases |
| S58 | MH tooth abnormalities+ |
| S57 | MH tooth wear+ |
| S56 | MH tooth wear+ |
| S55 | MH tooth injuries+ |
| S54 | MH tooth loss+ |
| S53 | MH tooth resorption+ |
| S52 | MH dental caries+ |
| S51 | MH tooth demineralization+ |
| S50 | MH periodontal disease+ |
| S49 | MH malocclusion+ |
| S48 | MH stomatognathic diseases |
| S47 | MH dental pulp diseases+ |
| S46 | MH cleft lip+ |
| S45 | MH Dental health services+ |
| S44 | MH toothache+ |
| S43 | MH Dental Plaque+ |
| S42 | Ti (dental hygiene or dental pulp exposure* or dental disease* or tooth abrasion* or tooth attrition* or dental wear* or tooth fracture* or tooth avulasion* or teeth injur* or dental carries or tooth hypomineralization or teeth hypomineralization or carious lesion* or root carries or dental fissure* or tooth mobilit* or tooth migration* or malocclusion* or tooth crowding* or cross bite* or oral health status or tooth birth defect* or dentistry or cleft lip or dental health service* or oral health program* or hair lip*) or ab (dental hygiene or dental pulp exposure* or dental disease* or tooth abrasion* or tooth attrition* or dental wear* or tooth fracture* or tooth avulasion* or teeth injur* or dental carries or tooth hypomineralization or teeth hypomineralization or carious lesion* or root carries or dental fissure* or tooth mobilit* or tooth migration* or malocclusion* or tooth crowding* or cross bite* or oral health status or tooth birth defect* or dentistry or cleft lip or dental health service* or oral health program* or hair lip*) |
| S41 | Ti (oral health or mouth disease* or oral hygien* or tooth disease* or tooth abnormal* or tooth wear* or tooth injur* or tooth loss* or tooth resorption or dental carries or tooth demineralization or periodontal disease* or stomatognathic disease* or dental pulp disease* or toothache* or plaque) or ab (oral health or mouth disease* or oral hygien* or tooth disease* or tooth abnormal* or tooth wear* or tooth injur* or tooth loss* or tooth resorption or dental carries or tooth demineralization or periodontal disease* or stomatognathic disease* or dental pulp disease* or toothache* or plaque) |
| S40 | (Ti (oral health or mouth disease* or oral hygien* or tooth disease* or tooth abnormal* or tooth wear* or tooth injur* or tooth loss* or tooth resorption or dental carries or tooth demineralization or periodontal disease* or stomatognathic disease* or dental pulp disease* or toothache* or plaque) or ab (oral health or mouth disease* or oral hygien* or tooth disease* or tooth abnormal* or tooth wear* or tooth injur* or tooth loss* or tooth resorption or dental carries or tooth demineralization or periodontal disease* or stomatognathic disease* or dental pulp disease* or toothache* or plaque)) AND (S62 OR S61 OR S60 OR S59 OR S58 OR S57 OR S56 OR S55 OR S54 OR S53 OR S52 OR S51 OR S50 OR S49 OR S48 OR S47 OR S46 OR S45 OR S44 OR S43 OR S42 OR S41) |
| S39 | S62 or S61 or S60 or S59 or S58 or S57 or S56 or S55 or S54 or S53 or S52 or S51 or S50 or S49 or S48 or S47 or S46 or S45 or S44 or S43 or S42 or S41 or S40 |
| S38 | (pediatric* or paediatric* or child* or newborn* or congenital* or infan* or baby or babies or neonat* or “pre-term” or preterm or “premature birth*” or NICU or preschool* or “pre-school*” or kindergarten* or “elementary school*” or “nursery school*” or schoolchild* or toddler* or boy or boys or girl* or “middle school*” or pubescen* or juvenile* or teen* or youth* or “high school*” or adolesc*or prepubesc* or “pre-pubesc*” or (MH "Child+") OR (MH "Adolescence+") OR (MH "Minors (Legal)") or "(MH "Child Abuse, Sexual") OR (MH "Child Behavior Disorders+") OR (MH "Child, Medically Fragile") OR (MH "Child Day Care") OR (MH "Child Behavior+") OR (MH "Child Mortality") OR (MH "Child Passenger Safety") OR (MH "Child Development Disorders, Pervasive+") OR (MH "Child Custody") OR (MH "Child Abuse+") OR (MH "Child Nutritional Physiology+") OR (MH "Child Behavior Checklist") ) OR SO ( child* or pediatric* or paediatric* or adolescent ) |
| S37 | MH United Arab Emirates |
| S36 | MH Arabs |
| S35 | TI (emirat* OR UAE OR bedouin* OR gulf OR (trucial N1 state*)) OR AB (emirat* OR UAE OR bedouin* OR gulf OR (trucial N1 state*)) |
| S34 | TI (dubai OR abudhabi OR (abu N1 dhabi) OR ajman OR fujaira* OR sharja* OR sharga* OR sharka* OR (ras N1 (alkhaima* OR elkhaima* OR ((al OR el) N1 khaima*))) OR ((um* OR om*) N1 ((al OR el) N1 (qaiwain OR quwain) OR alqaiwain OR alquwain OR elqaiwain OR elquwain)) OR AB (dubai OR abudhabi OR (abu N1 dhabi) OR ajman OR fujaira* OR sharja* OR sharga* OR sharka* OR (ras N1 (alkhaima* OR elkhaima* OR ((al OR el) N1 khaima*))) OR ((um* OR om*) N1 ((al OR el) N1 (qaiwain OR quwain) OR alqaiwain OR alquwain OR elqaiwain OR elquwain)) |
| S33 | S37 or S36 or S35 or S34 |
| S32 | S39 AND S38 AND S33 |
| S31 | S24 AND S25 AND S30 |
| S30 | S26 or S27 or S28 or S29 |
| S29 | TI (dubai OR abudhabi OR (abu N1 dhabi) OR ajman OR fujaira* OR sharja* OR sharga* OR sharka* OR (ras N1 (alkhaima* OR elkhaima* OR ((al OR el) N1 khaima*))) OR ((um* OR om*) N1 ((al OR el) N1 (qaiwain OR quwain) OR alqaiwain OR alquwain OR elqaiwain OR elquwain)) OR AB (dubai OR abudhabi OR (abu N1 dhabi) OR ajman OR fujaira* OR sharja* OR sharga* OR sharka* OR (ras N1 (alkhaima* OR elkhaima* OR ((al OR el) N1 khaima*))) OR ((um* OR om*) N1 ((al OR el) N1 (qaiwain OR quwain) OR alqaiwain OR alquwain OR elqaiwain OR elquwain)) |
| S28 | TI (emirat* OR UAE OR bedouin* OR gulf OR (trucial N1 state*)) OR AB (emirat* OR UAE OR bedouin* OR gulf OR (trucial N1 state*)) |
| S27 | MH Arabs |
| S26 | MH United Arab Emirates |
| S25 | (pediatric* or paediatric* or child* or newborn* or congenital* or infan* or baby or babies or neonat* or “pre-term” or preterm or “premature birth*” or NICU or preschool* or “pre-school*” or kindergarten* or “elementary school*” or “nursery school*” or schoolchild* or toddler* or boy or boys or girl* or “middle school*” or pubescen* or juvenile* or teen* or youth* or “high school*” or adolesc*or prepubesc* or “pre-pubesc*” or (MH "Child+") OR (MH "Adolescence+") OR (MH "Minors (Legal)") or "(MH "Child Abuse, Sexual") OR (MH "Child Behavior Disorders+") OR (MH "Child, Medically Fragile") OR (MH "Child Day Care") OR (MH "Child Behavior+") OR (MH "Child Mortality") OR (MH "Child Passenger Safety") OR (MH "Child Development Disorders, Pervasive+") OR (MH "Child Custody") OR (MH "Child Abuse+") OR (MH "Child Nutritional Physiology+") OR (MH "Child Behavior Checklist") ) OR SO ( child* or pediatric* or paediatric* or adolescent ) |
| S24 | S1 or S2 or S3 or S4 or S5 or S6 or S7 or S8 or S9 or S10 or S11 or S12 or S13 or S14 or S15 or S16 or S17 or S18 or S19 or S20 or S21 or S22 or S23 |
| S23 | (Ti (oral health or mouth disease* or oral hygien* or tooth disease* or tooth abnormal* or tooth wear* or tooth injur* or tooth loss* or tooth resorption or dental carries or tooth demineralization or periodontal disease* or stomatognathic disease* or dental pulp disease* or toothache* or plaque) or ab (oral health or mouth disease* or oral hygien* or tooth disease* or tooth abnormal* or tooth wear* or tooth injur* or tooth loss* or tooth resorption or dental carries or tooth demineralization or periodontal disease* or stomatognathic disease* or dental pulp disease* or toothache* or plaque)) AND (S1 OR S2 OR S3 OR S4 OR S5 OR S6 OR S7 OR S8 OR S9 OR S10 OR S11 OR S12 OR S13 OR S14 OR S15 OR S16 OR S17 OR S18 OR S19 OR S20 OR S21 OR S22) |
| S22 | Ti (oral health or mouth disease* or oral hygien* or tooth disease* or tooth abnormal* or tooth wear* or tooth injur* or tooth loss* or tooth resorption or dental carries or tooth demineralization or periodontal disease* or stomatognathic disease* or dental pulp disease* or toothache* or plaque) or ab (oral health or mouth disease* or oral hygien* or tooth disease* or tooth abnormal* or tooth wear* or tooth injur* or tooth loss* or tooth resorption or dental carries or tooth demineralization or periodontal disease* or stomatognathic disease* or dental pulp disease* or toothache* or plaque) |
| S21 | Ti (dental hygiene or dental pulp exposure* or dental disease* or tooth abrasion* or tooth attrition* or dental wear* or tooth fracture* or tooth avulasion* or teeth injur* or dental carries or tooth hypomineralization or teeth hypomineralization or carious lesion* or root carries or dental fissure* or tooth mobilit* or tooth migration* or malocclusion* or tooth crowding* or cross bite* or oral health status or tooth birth defect* or dentistry or cleft lip or dental health service* or oral health program* or hair lip*) or ab (dental hygiene or dental pulp exposure* or dental disease* or tooth abrasion* or tooth attrition* or dental wear* or tooth fracture* or tooth avulasion* or teeth injur* or dental carries or tooth hypomineralization or teeth hypomineralization or carious lesion* or root carries or dental fissure* or tooth mobilit* or tooth migration* or malocclusion* or tooth crowding* or cross bite* or oral health status or tooth birth defect* or dentistry or cleft lip or dental health service* or oral health program* or hair lip*) |
| S20 | MH Dental Plaque+ |
| S19 | MH toothache+ |
| S18 | MH Dental health services+ |
| S17 | MH cleft lip+ |
| S16 | MH dental pulp diseases+ |
| S15 | MH stomatognathic diseases |
| S14 | MH malocclusion+ |
| S13 | MH periodontal disease+ |
| S12 | MH tooth demineralization+ |
| S11 | MH dental caries+ |
| S10 | MH tooth resorption+ |
| S9 | MH tooth loss+ |
| S8 | MH tooth injuries+ |
| S7 | MH tooth wear+ |
| S6 | MH tooth wear+ |
| S5 | MH tooth abnormalities+ |
| S4 | MH tooth diseases |
| S3 | MH oral hygiene+ |
| S2 | MH mouth diseases |
| S1 | MH oral health+ |

**Cochrane Library**

ID Search

#1 MeSH descriptor: [Oral Health] explode all trees

#2 MeSH descriptor: [Mouth Diseases] this term only

#3 MeSH descriptor: [Oral Hygiene] explode all trees

#4 MeSH descriptor: [Tooth Diseases] this term only

#5 MeSH descriptor: [Tooth Abnormalities] explode all trees

#6 MeSH descriptor: [Tooth Wear] explode all trees

#7 MeSH descriptor: [Tooth Injuries] explode all trees

#8 MeSH descriptor: [Tooth Loss] explode all trees

#9 MeSH descriptor: [Tooth Resorption] explode all trees

#10 MeSH descriptor: [Tooth Demineralization] explode all trees

#11 MeSH descriptor: [Periodontal Diseases] explode all trees

#12 MeSH descriptor: [Malocclusion] explode all trees

#13 MeSH descriptor: [Stomatognathic Diseases] this term only

#14 MeSH descriptor: [Dental Pulp Diseases] explode all trees

#15 MeSH descriptor: [Cleft Lip] explode all trees

#16 MeSH descriptor: [Dental Health Services] explode all trees

#17 MeSH descriptor: [Toothache] explode all trees

#18 MeSH descriptor: [Dental Plaque] explode all trees

#19 (((tooth or teeth or dental or oral or mouth) NEAR/3 (health or disease? or hygien* or abnormalit* or wear* or injur* or loss or resorption? or fractur* or (birth NEAR defect?) or fissure? or anomalies or malformation* or fused))):ti,ab,kw (Word variations have been searched)

#20 (((periodental or stomatognathic) Near3 disease)):ti,ab,kw (Word variations have been searched)

#21 ((toothache* or dentistry or malocclusion* or (cleft NEAR/ lip*) or (cross NEAR/ bite*) or (hair NEAR/ lip*) or (root NEAR/ caries) or (carious NEAR/ lesion))):ti,ab,kw (Word variations have been searched)

#22 ((dental or tooth or teeth) NEAR2 (exposure? or abrasion* or attrition* or avulasion* or caries or demineralization? or hypomineralization* or plaque or migration* or crowding? or mobilit*))

#23 #1 or #2 or #3 or #4 or #5 or #6 or #7 or #8 or #9 or #10 or #11 or #12 or #13 or #14 or #15 or #16 or #17 or #18 or #19 or #20 or #21 or #22

#24 MeSH descriptor: [Adolescent] explode all trees

#25 MeSH descriptor: [Child] explode all trees

#26 MeSH descriptor: [Pediatric Dentistry] explode all trees

#27 ((pediatric* or paediatric* or child* or newborn* or congenital* or infan* or baby or babies or neonat* or pre term or preterm* or premature birth or NICU or preschool* or pre school* or kindergarten* or elementary school* or nursery school* or schoolchild* or toddler* or boy or boys or girl* or middle school* or pubescen* or juvenile* or teen* or youth* or high school* or adolesc* or prepubesc* or pre pubesc*)):ti,ab,kw (Word variations have been searched)

#28 #24 or #25 or #26 or #27

#29 MeSH descriptor: [United Arab Emirates] explode all trees

#30 MeSH descriptor: [Arabs] explode all trees

#31 ((emirat* or UAE or bedouin* or gulf or (trucial Near/ state*))):ti,ab,kw (Word variations have been searched)

#32 ((dubai or abudhabi or (abu NEAR/ dhabi) or ajman or fujaira* or sharja* or sharga* or sharka* or (ras NEAR (alkhaima* or elkhaima* or ((al or el) NEAR/ khaima*))) or ((um* or om*) NEAR (((al or el) NEAR/ (qaiwain or quwain)) or alqaiwain or alquwain or elqaiwain or elquwain)))):ti,ab,kw (Word variations have been searched)

#33 #29 #30 or #31 or #32

#34 #23 AND #28 AND #34

**IMEMR**

oral health or mouth disease* or oral hygien* or tooth disease* or tooth abnormal* or tooth wear* or tooth injur* or tooth loss* or tooth resorptionor dental carries or tooth demineralization or periodontal disease* or stomatognathic disease* or dental pulp disease* or toothache* or plaque or dental hygiene or dental pulp exposure* or dental disease* or tooth abrasion* or tooth attrition* or dental wear* or tooth fracture*or tooth avulasion* or teeth injur* or dental carries or tooth hypomineralization or teeth hypomineralization or carious lesion* or root carries or dental fissure* or tooth mobilit* or tooth migration* or malocclusion* or tooth crowding* or cross bite* or oral health status or tooth birth defect* or dentistry or cleft lip or dental health service* or oral health program*or hair lip*
